# Image directed lymph node sampling for lung cancer staging

**DOI:** 10.1186/1470-7330-14-S1-P32

**Published:** 2014-10-09

**Authors:** Leslie E Quint, Douglas Arenberg, Rishindra M Reddy, Jules Lin

**Affiliations:** 1 University of Michigan, Ann Arbor, MI, USA

## Educational poster objectives

• To display methods of regional lymph node sampling for staging bronchogenic cancer.

• To illustrate how imaging directs the optimal methods of sampling in individual patients.

## Introduction

• Summarize the lung cancer staging system.

• Show regional lymph node stations using diagrams and CT/PET images.

• Explain the need for tissue sampling in order to establish the highest stage and direct appropriate therapy. Sampling techniques with illustrations and imaging examples of accessible lymph nodes:

• Mediastinoscopy: Gold standard; outpatient surgical technique; optimal for stations 2, 4, and 7; tissue samples from all available nodal stations.

• Transbronchial needle aspiration (TBNA) with endobronchial ultrasound (EBUS): Optimal for stations 2, 4, 7, 10-12; often only abnormal nodes sampled; cytologic sample; least invasive; not universally available.

• Chamberlain procedure: Outpatient surgical technique; historical standard for stations 5 and 6; tissue samples.

• Video assisted thoracoscopic surgery (VATS): Surgical technique; optimal for stations 2R, 4R, 5, 6, 7, 8, 9, 10, and 11; tissue samples; supplanting Chamberlain procedure; must tolerate single lung ventilation.

• Endoscopic ultrasound (EUS): Optimal for stations 3p, 4L, 7, 8, 9, and occasionally 3a, 2R, 2L, 4R; cytologic sample; not universally available.

• CT guided biopsy: not routine; different approaches (e.g. parasternal) and patient positioning; optimal for large nodes; core biopsy.

## Summary

In patients with lung cancer, choosing a method of lymph node sampling depends on anatomy, availability of techniques, amount of tissue needed, cost, and safety. CT and PET imaging are crucial for depicting anatomical relationships and directing the appropriate sampling technique.

